# A Case Report of Plasmacytoma in a 28-Year-Old Patient: Bridging the Age Gap in a Rare Presentation

**DOI:** 10.7759/cureus.47671

**Published:** 2023-10-25

**Authors:** Naveen Kizhakkayil Tency, Julieanna A Camarena, Archa Roy, Mini Pradeep

**Affiliations:** 1 Department of Internal Medicine, Government T D Medical College, Alappuzha, IND; 2 Department of Internal Medicine, University of Texas Medical Branch, Galveston, USA

**Keywords:** compression fracture spine, delayed diagnosis, oncology, solitary bone plasmacytoma, young adult male

## Abstract

Plasmacytoma, an uncommon malignancy originating from plasma cells, predominantly manifests in the elderly demographic. However, its incidence among young adults remains infrequent. Herein, we present a case involving a 28-year-old young adult diagnosed with solitary bone plasmacytoma. The patient presented with acute exacerbation of chronic lower back pain of two years, which, upon hospitalization, was attributed to a lumbar spine compression fracture. Comprehensive blood analysis, imaging studies, and pathology assessments suggested the likelihood of solitary plasmacytoma, devoid of indicators characteristic of multiple myeloma (MM). The patient was given symptomatic treatment and underwent surgical spine decompression, followed by the commencement of radiation therapy to address the malignancy. Subsequent to radiotherapeutic intervention, a noteworthy amelioration in pain and overall condition was observed. This case report assumes importance due to the insight it provides into the natural progression of solitary plasmacytoma. Patients with pathological fractures warrant thorough assessment for solitary plasmacytoma, necessitating vigilant monitoring for its potential evolution into MM. This case serves as a pertinent illustration of the need to expand our existing knowledge of solitary bone plasmacytoma, moving beyond the conventional notion that it predominantly afflicts the elderly population.

## Introduction

Plasmacytoma is a rare plasma cell dyscrasia affecting the axial skeleton and soft tissue without any evidence of systemic manifestations. It is considered a precursor of plasma cell malignancies, such as multiple myeloma (MM) [1]. Plasmacytoma can be presented as solitary or multiple lesions anywhere in the body. The International Myeloma Working Group divides plasmacytoma into two: solitary plasmacytoma of the bone (SPB) and extramedullary plasmacytoma (EMP). Multiple solitary plasmacytoma is an even rare presentation that consists of monoclonal cell infiltrates in one or more lytic bone lesions or extramedullary tissue. Solitary plasmacytoma occurs more commonly in men than in women (65% vs. 35%), and the median age is 55 years. The most common symptom at presentation is pain at the site of the skeletal lesion [2]. Plasmacytoma is commonly managed with surgery, radiotherapy, and chemotherapy as required. Therapy for local disease gives prolonged survival, and disseminated disease treatment gives longer remission [3]. This case report presents a case of SPB in a young patient and provides insight into the incidence of such a rare malignancy in the younger population and its evaluation and further follow-up.

## Case presentation

A 28-year-old male patient presented with an acute exacerbation of chronic lower back pain of a two-year duration. The patient had intermittent radiation of pain to the proximal aspect of his anterior thigh but no numbness or bowel/bladder dysfunction. He was not on any medications previously for the pain. He denied any history of trauma, fever, or weight loss. The pain was relieved upon taking IV morphine 5 mg Q4H as needed and oral oxycodone 30 mg Q4H in the emergency room. Physical examination revealed diffuse tenderness over the lumbar spine, pain limiting flexion and extension of the lower limbs, and brisk plantar reflex. There were no neurological deficits or skin findings. The patient did not have any significant past medical or family history of malignancies or chronic infections and no history of any drug abuse.

The initial laboratory investigations are summarized in Table 1. Additionally, AFB and QuantiFERON were negative. Vitamin D level was low, and parathyroid hormone and thyroid-stimulating hormone were all normal. A blood peripheral smear revealed leukocytosis with absolute neutrophilia, monocytosis, eosinopenia, no blast cells, and giant platelets. This helped rule out possible etiologies like spinal abscesses, spinal tuberculosis, osteomyelitis, and hyperparathyroidism.

**Table 1:** Relevant initial laboratory findings H: high value, HGB: hemoglobin, WBC: white blood cell count, ESR: erythrocyte sedimentation rate, CRP: C-reactive protein, BUN: blood urea nitrogen, LDH: lactate dehydrogenase, IgA: immunoglobulin A, IgG: immunoglobulin G, IgM: immunoglobulin M

Parameter	Test result	Normal range
HGB	15.4	12.2-16.4 g/dl
WBC	12.53 (H)	4.20-10.70 10^3/μL
Platelet count	208	150-328 10^3/μL
ESR	46 (H)	2-30 mm/hr
CRP	8.0 (H)	<0.8 mg/dl
Creatinine	0.83	0.7-1.2 mg/dl
BUN	16	6-20 mg/dl
Total protein	6.4	6.3-8.2 g/dl
Alkaline phosphatase	71	34-122 U/L
LDH	481 (H)	120-246 U/L
Calcium	8.8	8.6-10.6 mg/dl
Beta 2 microglobulin	1.6	0.8-2.4 mg/L
Alpha 1	0.3	0.2-0.4 g/dl
Alpha 2	0.6	0.6-1.2 g/dl
Beta	1.0	07-1.4 g/dl
Gamma	0.9	1.0-1.8 g/dl
IgA	254	70-312 mg/dl
IgG	859	636-1600 mg/dl
IgM	137	56-352 mg/dl
Kappa Qnt free light chains	32.38 (H)	3.30-19.40 mg/L
Lambda Qnt free light chain	25.09	5.71-26.30 mg/L
Kappa/lambda free light chain ratio	1.29	0.26-1.65

Subsequent investigation showed serum protein electrophoresis and urine protein electrophoresis to be abnormal, with an M spike of 0.6 (normal 0-0.001 g/dl) and immunofixation electrophoresis with IgG lambda type monoclonal gammopathy. The B cells were polyclonal with no co-expression of CD5 or CD10. The CD4:CD8 ratio was about 0.6:1. Polyclonal plasma cells without any antigenic aberrancy are present at 0.6% of all events analyzed. There were no increased events in the CD45 dim blast gate. Positive markers identified were CD 19, 138, 38, 27, and 81.

**Figure 1:**
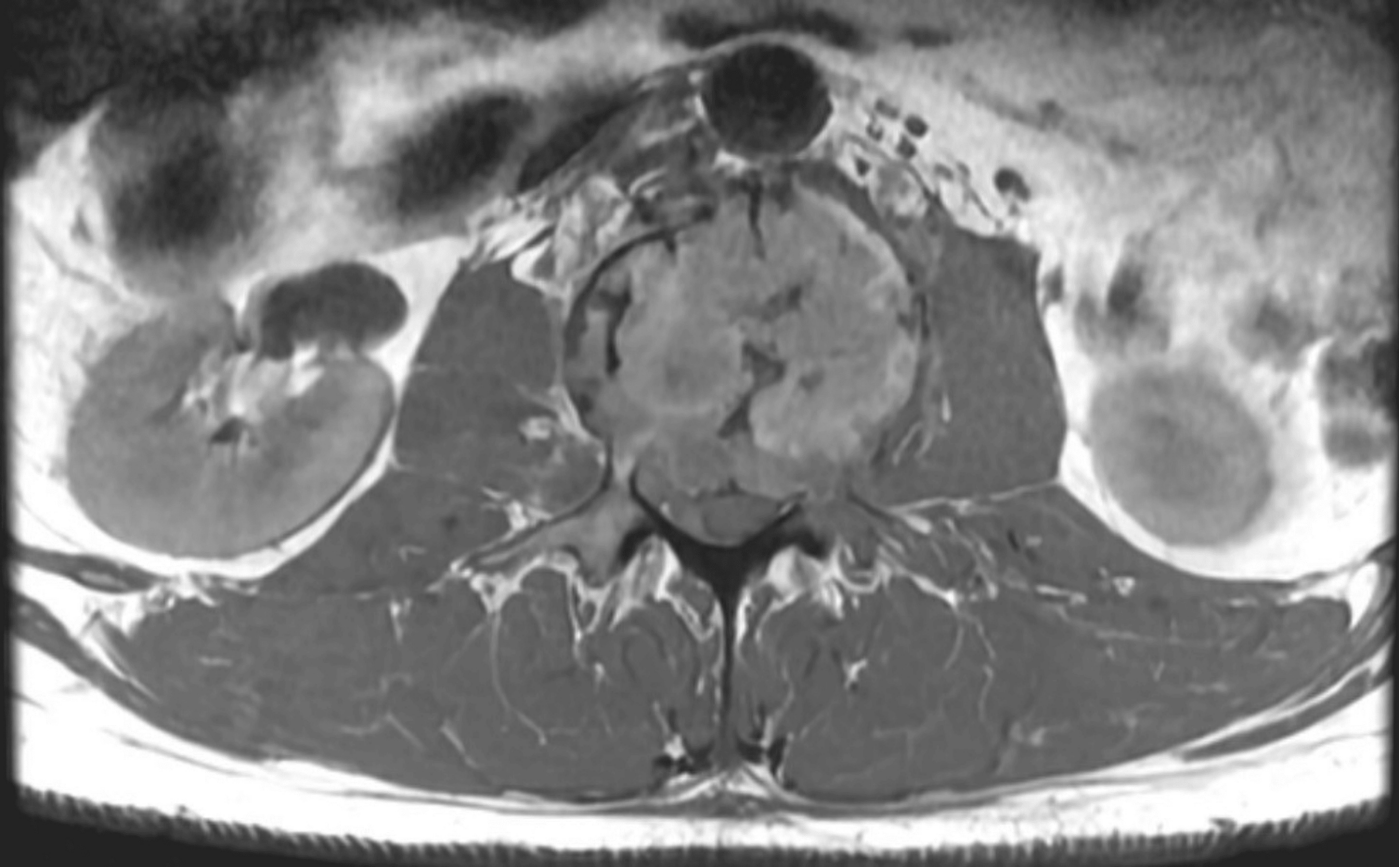
Axial section of MRI at the level of L2 vertebrae showing destructive changes of L2 vertebrae MRI: magnetic resonance imaging, L2: second lumbar vertebrae

**Figure 2:**
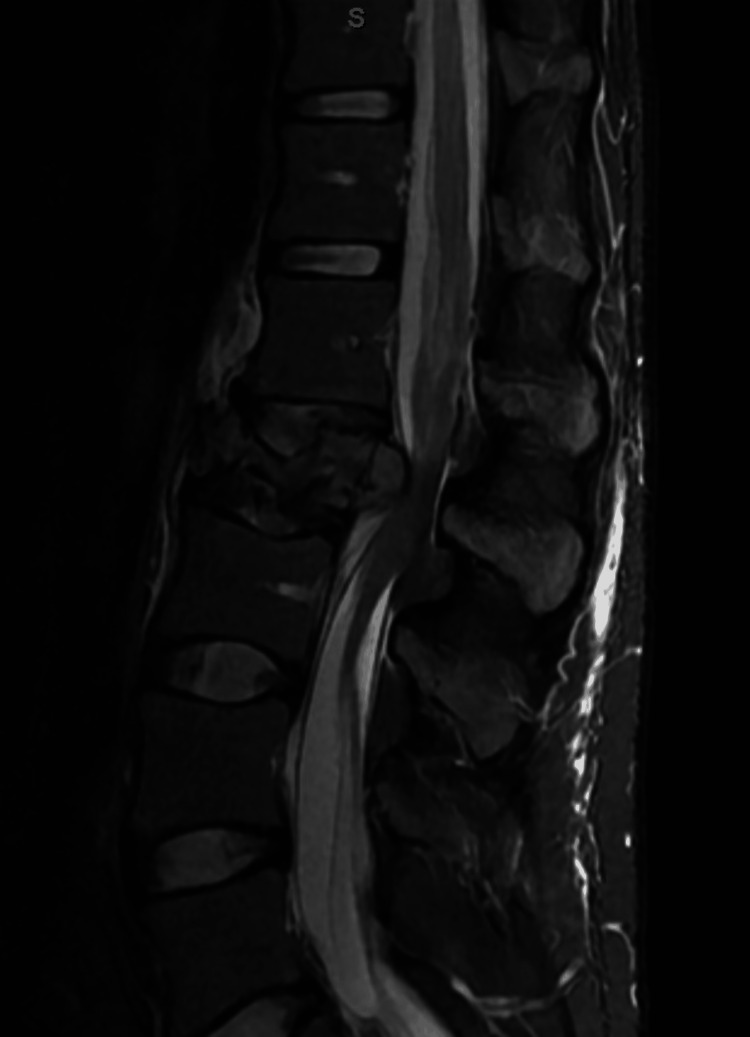
Sagittal section of lumbar spine MRI showing fracture of L2 vertebrae with narrowing of the spinal canal and compression of the cauda equina nerve roots MRI: magnetic resonance imaging, L2: second lumbar vertebrae

The CT scan of the lumbar and thoracic spine without contrast shows destructive changes in the L2 vertebral body. The MRI of the lumbar spine with and without contrast underscored a severe burst fracture at the L2 vertebral body (Figure 1). This fracture has caused a drastic loss in vertebral height, estimated to be around 90%, leading to a substantial narrowing of the spinal canal and compression of the cauda equina nerve roots (Figure 2). Moreover, anterior lateral epidural soft tissues were thickened and enhanced at the L1-L2 level. These findings collectively raise a high degree of concern for a pathologic fracture induced by an underlying neoplastic process. No other lesions were detected. Suspicion of enlarged para-aortic lymph nodes was also mentioned. CT with contrast of head and thorax were normal except for some prominent chronic pulmonary nodularity.

**Figure 3:**
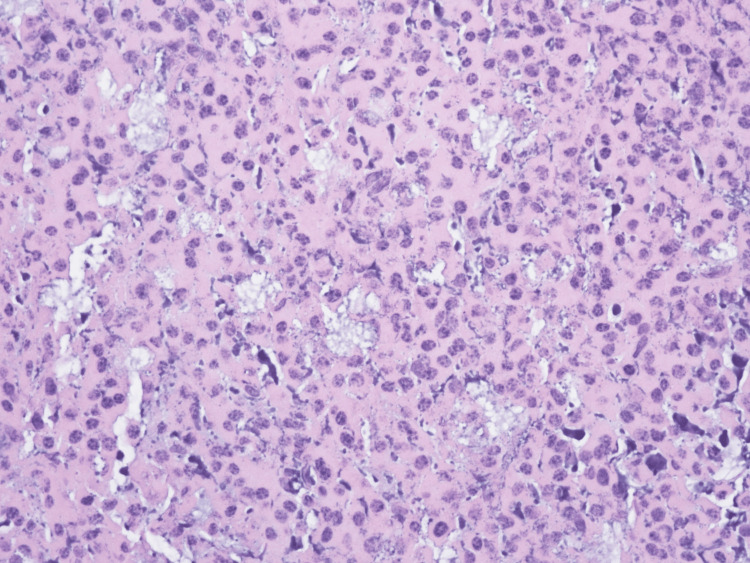
Soft tissue and epidural mass pathology Extensively necrotic fibroconnective tissue with plasmacytic infiltrates

He was initially managed symptomatically for the back pain with oral Tylenol 1000 mg Q6H, oral oxycodone 10 mg Q4H, oral methocarbamol 500 mg Q6H, and, as needed, IV morphine 5 mg. For the fracture of the lumbar spine, surgical intervention with T11-L4 instrumentation and fusion, L1-L3 laminectomies for decompression, right L2 transpedicular decompression, and posterolateral arthrodesis with bone graft material from T11-L4 were performed along with tissue biopsy of tumor region. The biopsy pathology showed a soft tissue mass with plasmacytic infiltrate and extensive necrotic fibroconnective tissue (Figure 3), which was non-specific, and, hence, a differential diagnosis of solitary plasmacytoma, MM, plasmablastic lymphoma, chronic infection, and reactive plasmacytosis was made. Bone marrow revealed normocellularity with progressive trilineage hematopoiesis and no morphologic or immunophenotypic support for plasma cell neoplasm, myeloid neoplasm, or lymphoma.

**Figure 4:**
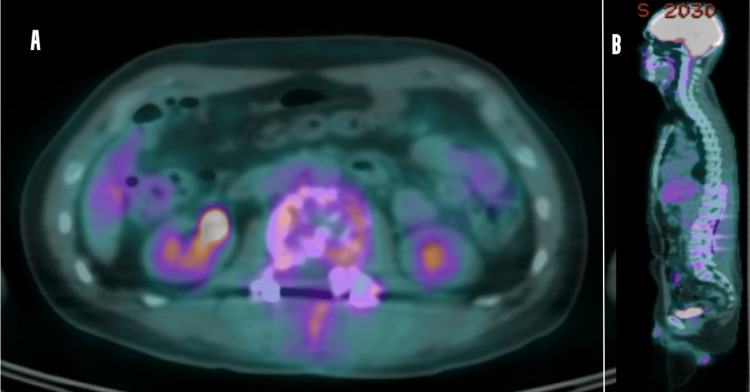
PET scan (A) axial section showing hypermetabolic lesion at the level of L2 vertebral level with reactive lymph nodes and (B) sagittal view showing solitary hypermetabolic lesion at the level of L2 vertebral level PET: positron emission tomography, L2: second lumbar vertebrae

Subsequently, sPET-CT scanning confirmed the presence of a solitary hypermetabolic lesion at the L2 vertebral level, along with a reactive lymph node (Figure 4A-4B). This ruled out any extramedullary manifestations or metastatic origin. Consequently, a clinical diagnosis of solitary plasmacytoma was made after ruling out MM, as none of the CRAB manifestations (hypercalcemia, renal insufficiency, anemia, and multiple bone lesions) were met. After discussing with the patient, the radiation-oncology team decided to start him on radiation therapy.

His radiation therapy commenced with a planned dose of 200 cGy per fraction, totaling 4400 cGy over 22 fractions. He tolerated radiation therapy well, except for mucositis. Pain management was continued during the radiotherapy. While he continued to experience radiating pain in his lower back, he denied any pain or numbness in his legs. After completing his radiation therapy, he reported a significant reduction in lower back pain, and he was feeling generally well. The repeat lab reports indicated that both serum and urine electrophoresis and immunofixation were within normal range, without the presence of an M spike. Further recommendations include retesting serum electrophoresis in six to eight weeks if clinically necessary. The patient is awaiting a follow-up visit.

## Discussion

SPB is a rare plasma cell disorder that accounts for 2% to 5% of all plasma cell malignancies. It is prevalent in older individuals with a mean age of 55 to 60 years, African-American race, and males (M:F 2:1) [4]. The exact cause of solitary bone plasmacytoma remains unknown. Inhaled irritants and viral infections are often linked to nasopharyngeal EMP. Plasmacytomas, arising in bone marrow (SPB) or mucosal cells (EMP), share traits with plasma disorders. IL-6 drives their growth, genetic anomalies (chromosome gains/losses) contribute, and high-grade cytology/angiogenesis speeds up the MM risk [5]. SPB is commonly seen in the axial skeleton with high red bone marrow, such as the ribs, vertebrae (thoracic more than lumber and cervical), femur, and pelvis [2]. SPB has been reported in uncommon sites like the skull (with headaches, diplopia, strabismus, exophytic mass, otalgia, and dizziness) [6], calcaneus [7], and jaw [8].

Patients with SPB typically present with pain as a result of bone destruction, spinal cord, and/or nerve root involvement. The specific signs and symptoms depend on the location of the bone involvement and the presence of neurological signs. The pain can intensify with movement and can sometimes be alleviated with pain medication [4]. SPB is diagnosed when there is a solitary lesion of the bone, which is confirmed by contrast CT or MRI, and has a biopsy-proven plasma cell infiltration, the bone marrow biopsy yields a normal result (<10% plasma cells), and there is an absence of myeloma-related organ dysfunction like CRAB criteria (hypercalcemia, renal insufficiency, anemia, and multiple bone lesions) [9].

Radiotherapy can be employed to eliminate the localized lesion due to the high radiosensitivity of the cells; hence, for SPB, radiotherapy is the preferred treatment option, which is observed to achieve an 80% control [3]. In cases where plasmacytomas occur in specific sites like the spine, leading to neurological issues or causing structural instability, surgery is the recommended approach. The role of chemotherapy in managing this condition remains a topic of debate [3,9,8]. Across numerous studies, chemotherapy demonstrates limited efficacy in terms of disease control or the prevention of complications. While chemotherapy does not reduce the likelihood of plasmacytoma progressing to MM, it does extend the time it takes for this progression to occur [3,9]. Autologous stem cell transplantation (ASCT) is another modality recommended for high-risk, recurrent, or extensive solitary plasmacytoma cases. Trials by Jantunen et al. targeting patients with recurrent SPB have yielded promising results in the past [10].

Though in our patient chemotherapy was not employed, it has been evidenced that systemic therapies have significantly enhanced survival rates for plasmacytoma patients alongside surgical options. In the 1990s, those under 65 often underwent high-dose melphalan followed by ASCT. Thalidomide gained traction from 2001 to 2008 but was discontinued due to side effects, giving way to lenalidomide. Bortezomib's approval in 2005 offered a new treatment avenue [11]. VAD (vincristine, adriamycin, dexamethasone) and second-generation proteasome inhibitors, like carfilzomib and ixazomib, gained approval alongside daratumumab and panobinostat [12]. Additional drugs, such as pomalidomide, daratumumab, and panobinostat, also received approval during the same period [13]. Adjuvant treatment aligned with evolving guidelines, while lenalidomide mainly served as second-line therapy.

Plasmacytoma can lead to various complications, such as bone pain, compression fractures, nerve damage, spinal injury, POEMS syndrome (polyneuropathy, organomegaly, endocrinopathy, MM, and skin changes), immunodeficiency, renal impairment, gastrointestinal bleeding, and drug toxicity related to treatment [8,14-16].

Plasmacytoma demands careful differentiation from other diseases due to shared characteristics. The differential diagnosis encompasses several conditions. MM is distinct through CRAB manifestations, numerous bone lesions, and monoclonal proteins in serum or urine [9,17]. Non-Hodgkin lymphoma can resemble plasmacytoma, but immunophenotyping aids differentiation based on CD19 and CD45 expression [18]. Reactive plasmacytosis lacks the light chain restriction observed in plasmacytoma, while plasmablastic lymphoma, often seen in immunosuppressed individuals, displays unique features like plasmablastic morphology and an association with the Epstein-Barr virus. Clarifying these distinctions is crucial for accurate diagnosis and treatment [19].

Around half of patients diagnosed with solitary plasmacytoma achieve a 10-year survival, with 25-40% experiencing a disease-free state at the same interval. More than 65% of these cases eventually develop into overt MM. The progression can occur as late as 15 years after the initial diagnosis [2]. Poor prognostic factors for SPB include age over 40, tumor size ≥5cm, spine lesions, neurological symptoms, high serum M protein levels, light chains presence, persistent M proteins after treatment, bone marrow infiltration, and osteopenia [9]. It's important to note that our case may exhibit some, but not necessarily all, of these indicators.

Patients treated for plasmacytoma need lifelong follow-up as there can be a recurrence or development of MM [2]. The National Comprehensive Cancer Network (NCCN) advises three to six-month follow-up with tests like blood count, chemistry, immunoglobulins, protein analysis, and urine tests. Additional tests like lactate dehydrogenase, beta-2 microglobulin, bone marrow, and imaging are done as needed. Yearly imaging for plasmacytomas is also recommended for five years [20].

This case study underscores the importance of broadening our comprehension of the demographic characteristics and clinical presentations associated with solitary bone plasmacytoma, challenging the prevailing assumptions that it primarily affects elderly individuals. Educating healthcare providers and patients about the potential diagnosis in young individuals is essential to bridge the knowledge gap. Given the absence of a known etiology, it is essential to explore potential factors contributing to the incidence of this substantial medical condition. Moving forward, there is a pressing need for extensive research aimed at elucidating the origins of solitary bone plasmacytoma, especially in the young population. In this case, we have followed the existing guidelines for the treatment, diagnosis, and follow-up. However, future research initiatives may prioritize the integration of advanced imaging methodologies, immunophenotyping, and genetic profiling. These advancements are pivotal for improving diagnostic precision and refining long-term follow-up protocols, thereby enhancing the overall management of this complex disease.

## Conclusions

SPB, while infrequent, presents as a noteworthy malignancy affecting the younger demographic. This case report underscores the importance of including SPB as a differential diagnosis, even among younger patients displaying pathological fractures. The essence of early recognition and subsequent therapeutic considerations like radiotherapy is emphasized by this report, encompassing discussions on existing prognostic determinants and the requisite guidelines for vigilant monitoring aimed at detecting potential progression from SPB to MM.

The scarcity of occurrences of this condition within the youthful population increases the challenges in formulating evidence-based treatment strategies and suitable follow-up regimens for this specific age cohort. Looking forward, a deeper understanding of etiologies and clinical behaviors in this cohort will lead to enhanced management strategies. Incorporating advanced diagnostic techniques in future research is imperative for precise diagnosis and improved care.
