# Correction: A Case Report of Plasmacytoma in a 28-Year-Old Patient: Bridging the Age Gap in a Rare Presentation

**DOI:** 10.7759/cureus.c414

**Published:** 2026-03-18

**Authors:** Naveen Kizhakkayil Tency, Julieanna A Camarena, Archa Roy, Mini Pradeep

**Affiliations:** 1 Department of Internal Medicine, Government T D Medical College, Alappuzha, IND; 2 Department of Internal Medicine, University of Texas Medical Branch, Galveston, USA

This article has been corrected to fix a minor typo in Table 1. IgG is listed as 859 and is flagged as “(H)”, while the reference range provided is 636–1600 mg/dL. The "(H)" flag has now been removed.

